# Common oxytocin polymorphisms interact with maternal verbal aggression in early infancy impacting blood pressure at age 5-6: The ABCD study

**DOI:** 10.1371/journal.pone.0216035

**Published:** 2019-06-24

**Authors:** Laetitia J. C. A. Smarius, Thea G. A. Strieder, Theo A. H. Doreleijers, Tanja G. M. Vrijkotte, M. H. Zafarmand, Susanne R. de Rooij

**Affiliations:** 1 Department of Public Health, Amsterdam Public Health Research Institute, Academic Medical Center, University of Amsterdam, Amsterdam, The Netherlands; 2 Academic Center for Child and Adolescent Psychiatry de Bascule, Amsterdam, The Netherlands; 3 Department of Child and Adolescent Psychiatry, VU University Medical Center, Amsterdam, The Netherlands; 4 Arkin Institute for Mental Health, Amsterdam, The Netherlands; 5 Department of Clinical Epidemiology, Biostatistics and Bio-informatics, Academic Medical Center, University of Amsterdam, Amsterdam, The Netherlands; University of South Florida, UNITED STATES

## Abstract

Early life stress has been shown to contribute to alterations in biobehavioral regulation. Genetic make-up, especially related to social sensitivity, might affect the child’s vulnerability to these alterations. This study examined whether maternal verbally aggressive behavior in early infancy interacts with oxytocin polymorphisms in changing resting cardiovascular outcomes at age 5–6. In the Amsterdam-Born-Children-and-their-Development-(ABCD)-study, a large prospective, observational, population-based birth cohort, maternal verbally aggressive behavior was assessed in the 13^th^ postnatal week (range 11–25 weeks, SD 2 weeks) by a questionnaire (maternal self-report). Indicators of resting cardiac autonomic nervous system activity (sympathetic drive by pre-ejection period, parasympathetic drive by respiratory sinus arrhythmia), heart rate, and blood pressure were measured at age 5–6 years. Data on oxytocin receptor gene polymorphisms *rs53576*, *rs2268498* and oxytocin polymorphisms *rs2740210*, *rs4813627*, were collected (N = 966 included). If the child was carrier of the *rs53576 GG* variant, exposure to maternal verbally aggressive behavior (10.6%) was associated with increased systolic blood pressure at age 5–6 (B = 4.9 mmHg,95% CI[2.2;7.7]). If the child was carrier of the *rs2268498 TT/TC* variant, exposure to maternal verbally aggressive behavior was associated with increased systolic blood pressure at age 5–6 (B = 3.0 mmHg,95%CI[1.0:5.0]). No significant interactions of maternal verbally aggressive behavior with oxytocin gene polymorphisms on heart rate or cardiac autonomic nervous system activity were found. In conclusion, oxytocin receptor gene polymorphisms may partly determine a child’s vulnerability to develop increased systolic blood pressure after being exposed to maternal verbally aggressive behavior in early infancy.

## Introduction

Infancy is a highly critical period for brain development. During the first months of life, essential brain structures are evolving and in basic structures synaptogenesis is accelerating [1]. Exposure to a single, but substantial, stressor during this early period might impact brain development and programming of the stress systems significantly. For instance, maternal report of interparental conflict has been positively associated with infants' activation in brain regions related to emotion processing and stress regulation (anterior cingulate cortex, caudate nucleus (part of dorsal striatum), hypothalamus, and thalamus) in response to the sound of their mothers’ angry voice at age 6–12 months [2], implying programming effects. Of note, between 2–4 months of age, synaptogenesis in the striatum is most rapid and total gray matter volume of the striatum reaches adult size at about 4 months of age [1], emphasizing the potential impact of stress exposure in this period of life.

Maternal verbally aggressive behavior in early infancy can be considered a profound early life stressor, as soothing maternal behavior would be more appropriate at this young age. We have recently shown that exposure to maternal verbally aggressive behavior at the age of 3 months is associated with increased systolic blood pressure (SBP) at age 5–6 years [3]. However, some children might be more vulnerable to maternal verbally aggressive behavior than others. Differences in vulnerability or resilience to stress are related to protective environmental factors, including parental and non-parental support, and differences in genetic profile [4]. The extent to which maternal verbally aggressive behavior in infancy is perceived as stressful and thereby potentially affects cardiovascular outcomes, might partly depend on the infant’s genetic make-up.

Single nucleotide polymorphisms (SNPs) in the oxytocin (*OXT*) gene and in the oxytocin receptor (*OXTR*) gene are potential candidates for gene-environment (GxE) interaction due to their differential associations with social sensitivity. Oxytocin is a neuropeptide, predominantly expressed in magnocellular neurons in the hypothalamic paraventricular (PVN) and supraoptic (SON) nuclei [5], which has key functions in childbirth, maternal behavior, social behavior and cardioprotection. Cardioprotective activities of oxytocin are lowering of blood pressure (BP), parasympathetic neuromodulation, anti-inflammatory activity and vasodilatation [6,7]. In adults, oxytocin has been shown to be involved in emotion recognition [8] and mentalizing, which is the ability to represent mental states of oneself and others [9]. An important component of mentalizing is ‘seeing leads to knowing’, a prerequisite of the development of empathic abilities present in non-human primates [10]. Although infants do not have complete mentalizing and empathic abilities yet, naturally occurring variations in social sensitivity due to *OXT* and *OXTR* gene polymorphisms may affect the infant’s perception of the social environment.

A number of different *OXT* and *OXTR* polymorphisms associated with social sensitivity, such as facial emotion recognition or vocalizing, could be relevant in the context of maternal verbally aggressive behavior. At first, a SNP located in the third intron of the *OXTR* gene, *rs53576*, involving a guanine (*G*) to adenine (*A*) substitution, has been associated with diminished prosocial features. Individuals with the *GG* genotype (i.e. the absence of the polymorphism or wild type) display beneficial prosocial traits including enhanced trust, self-esteem and empathic abilities [11]. Recently, the *rs53576 G*-allele has been associated with increased amygdala responsiveness to emotional facial expressions [12], implicating a higher level of arousal, if exposed to emotional facial expressions. Interestingly, young adult *GG* carriers were demonstrated to be more sensitive to the social stressor of rejection as indicated by increased stress reactivity of BP and cortisol levels [13]. Because *G-*carriers are potentially more socially sensitive, this may render them more vulnerable to a negative social stressor. The second *OXTR* polymorphism of interest is *rs2268498*, upstream variant 2KB, located in the promoter region of the *OXTR* gene. *T-*allele carriers, both the wild homozygote and the heterozygote type, have been shown to perform better at facial emotion recognition [14] and seem to have higher self-reported empathy [15,16], compared to *C-*allele carriers.

Additionally, two particular *OXT* polymorphisms, *rs2740210*, located in the 3′ flanking region, upstream variant 2KB, and *rs4813627*, located 56kb downstream the *OXT* gene, have been found associated with the duration of maternal infant-directed vocalizing, a measure of maternal engagement [17]. It is not known what the function of these polymorphisms is in infants, but it could be hypothesized that *rs2740210* and *rs4813627* are related to infant’s social sensitivity.

**Fig 1:**
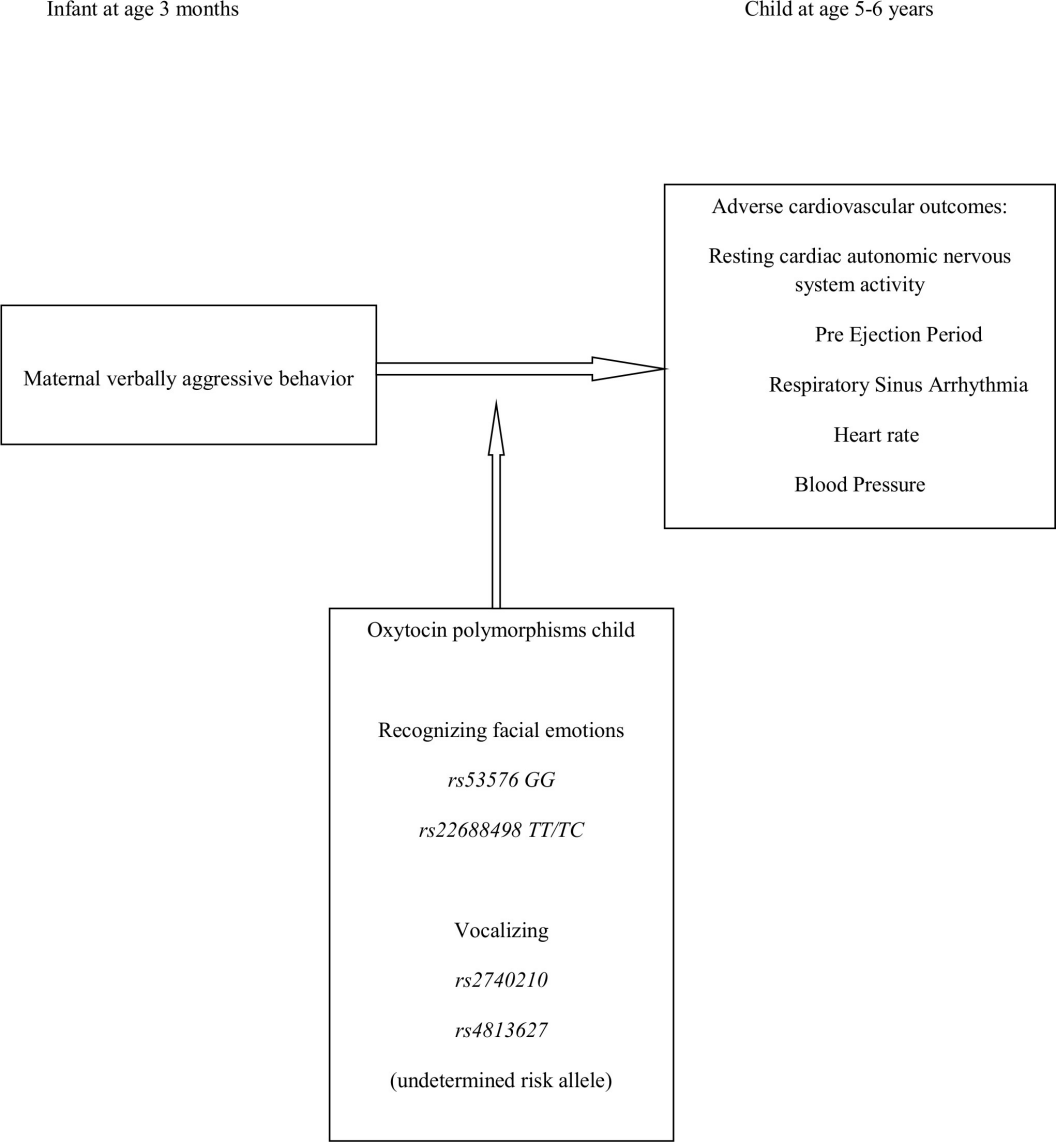
Hypothesis.

Ample evidence has shown that stressful experiences early in life can increase the risk of cardiovascular health problems and diseases in later life through programming of the biobehavioral response to stress, with altered activity of the stress systems, including the hypothalamic-pituitary-adrenal axis and the autonomic nervous system [18,19,20,21]. In line with the findings from the McQuaid et al. study[13], we hypothesize that individuals carrying *OXTR* and *OXT* SNPs enhancing social sensitivity are more vulnerable to a negative social stressor and display increased stress responses programming biobehavioural regulation with adverse cardiovascular consequences on the long term. Therefore, we aimed to test whether our previously demonstrated association between exposure to maternal verbally aggressive behavior in early infancy and increased SBP in children at age 5–6 [3] is moderated by *OXTR* and *OXT* SNPs. In addition, we included the cardiovascular outcomes heart rate (HR) and resting autonomic nervous system (ANS) activity at age 5–6. We expected that infants carrying *OXTR rs53576* GG or *rs2268498 T-*allele, who are more apt at recognizing facial emotions, experience more stress in response to maternal verbally aggressive behavior and display more adverse cardiovascular outcomes at age 5–6, compared to infants carrying respectively *GA/AA* or *CC* variants. Additionally, we explored whether carriers of one of the different variants of *OXT rs2740210* or *rs4813627* are more or less vulnerable to adverse cardiovascular outcomes after experiencing maternal verbally aggressive behavior in infancy, compared to carriers of the other variant. As these *OXT* variants were only investigated in mothers and shown to be associated with maternal duration of vocalizing, of which it is not known which duration is related to social sensitivity, we did not hypothesize beforehand which allele of *OXT rs2740210* or *rs4813627* would be the risk allele for social sensitivity. Our hypotheses are visualized in Fig 1.

## Results

### Subjects’ characteristics

The characteristics of both the mothers and the children are presented in Table 1. Of the 966 included children, 102 (10.6%) had been exposed to maternal verbally aggressive behavior at the age of 3 months (mean 13^th^ week, range 11–25 weeks, SD 2 weeks). Compared to children not exposed to maternal verbally aggressive behaviour, exposed children were slightly taller, had a mother who reported more postnatal depressive symptoms, less pleasure in infant care and more physically aggressive behaviour. When the children were 5–6, their mothers reported more depressive symptoms, more parenting stress, and more often used an authoritarian parenting style, compared to the mothers from the non-exposed children.

**Table 1:** Characteristics of 966 native Dutch women and their children according to maternal verbally aggressive behavior in early infancy (N = 102).

	N 966	Full sample % or mean (SD)	No maternal verbally aggressive behavior N = 864 (89.4%)	Maternal verbally aggressive behavior N = 102 (10.6%)	*p*
*Child characteristics*					
Gender: female	501	51.9	52.0	51.0	0.466
Birth weight (grams)	966	3567 (479)	3566 (481)	3622 (479)	0.222
Gestational age (wks)	966	39.7 (1.2)	39.7 (1.2)	39.7 (1.2)	0.991
Excessive crying	17	1.8	1.6	2.9	0.264
*Maternal characteristics*					
Pre-pregnancy BMI (kg/m2)	966	22.79 (3.48)	22.79 (3.49)	22.76 (3.38)	0.909
Pre-pregnancy hypertension	29	3	3	2.9	0.632
Pregnancy related hypertension	89	9.3	8.9	12.7	0.137
Primipara	536	55.5	55.0	59.8	0.206
Maternal age (years)	966	33.05 (4.99)	33.12 (3.98)	32.49 (4.07)	0.131
Cohabitancy: living with partner	896	92.9	92.9	93.1	0.579
Education, years after primary school	964	10.8 (2.9)	10.8 (2.9)	10.5 (3.0)	0.288
*Maternal factors in infancy*					
Depression (CES-D)	965	7.8 (6.3)	7.4 (6.1)	11.1 (7.1)	<0.001
Pleasure in infant care	961	8.7 (1.2)	8.6 (1.1)	9.1 (1.5)	<0.001
Smoking at home	39	4.0	4.3	2.0	0.199
Physical aggressive behavior	966	3.0 (0.2)	3.0 (0.1)	3.2 (0.5)	<0.001
*Child characteristics at 5–6 years*					
Intact family at child age 5–6	862	89.4	89.9	85.3	0.106
Age of the child (years)	963	5.12 (0.20)	5.12 (0.20)	5.10 (0.17)	0.210
Height at age 5 (cms)	966	115.32 (5.26)	115.17 (5.30)	116.53 (4.67)	0.014
BMI at age 5 (kg/m2) Time of day of measurements	966 915	15.34 (1.29) 1.55 (0.65)	15.33 (1.31) 1.54 (0.64)	15.43 (1.09) 1.60 (0.69)	0.478 0.421
*Maternal characteristics at the child’s age 5–6*					
Authoritarian Parenting Style	936	4.75 (2.59)	4.69 (2.56)	5.23 (2.78)	0.048
Maternal depressive symptoms	936	0.88 (1.78)	0.81 (1.68)	1.49 (2.41)	0.000
Parenting Stress (NOSI-K)	928	11.5 (2.7)	11.5 (2.7)	12.2 (3.0)	0.014
*Family hypertension*					
Paternal hypertension	44	4.6	4.9	2.0	0.136
Family (M or P family) hypertension	53	5.7	5.4	8.0	0.196

M = maternal; P = paternal; CES-D = Centre for Epidemiologic Studies Depression Scale; NOSI-K = ‘Nijmeegse Ouderlijke Stress Index’ for children. BMI = Body Mass Index

Time of day of measurements: 1 = 08:30am-10:30am; 2 = 10:30am-12:30pm; 3 = 12:30pm-15:00pm.

*Pleasure in infant care: Lower ratings of pleasure in infant care are corresponding with higher levels of Pleasure in infant care

### Maternal verbally aggressive behavior in infancy and child’s *OXTR* and *OXT* genotype variants

Maternal verbally aggressive behavior in infancy was not associated with any *OXTR* and *OXT* genotype variant of the infant (Table 2).

**Table 2:** *OXTR* and *OXT* genotype variants according to maternal verbally aggressive behavior, BP, resting HR and Cardiac ANS activity in sitting position.

	N (%)	Maternal verbally aggressive behavior No Yes		SBP (mmHg) Mean (SD)	DBP (mmHg) Mean (SD)	HR (bpm) Mean (SD)	RSA (msec) Mean (SD)	PEP (msec) Mean (SD)
		N (%)	N (%)					
*rs53576* GG (wild type)	403 (41.7)	363 (42.0)	40 (39.2)	96.3 (8.2)	56.9 (7.1)	91.7 (9.8)	110.3 (53.9)	71.9 (10.9)
GA	458 (47.4)	412 (47.7)	46 (45.1)	96.6 (8.4)	56.8 (6.9)	92.2 (9.5)	108.1 (51.4)	72.8 (11.5)
AA	105 (10.9)	89 (10.3)	16 (15.7)	95.7 (8.1)	55.4 (6.8)	90.7 (9.3)	109.0 (49.8)	73.6 (12.2)
*rs2268498* TT (wild type)	286 (29.6)	259 (30)	27 (26.5)	95.3 (7.9)	56.3 (7.2)	90.6 (9.5)	112.7 (50.8)	72.0 (10.9)
TC	486 (50.3)	430 (49.8)	56 (54.9)	97.2 (8.6)**	57.2 (6.9)	92.9 (9.6)**	108.4 (54.1)	72.2 (11.4)
CC	194 (20.1)	175 (20.3)	19 (18.6)	95.9 (7.7)	56.1 (6.6)	90.9 (9.4)	105.6 (49.6)	73.9 (11.9)
*rs2740210* CC (wild type)	479 (49.6)	421 (48.7)	58 (56.9)	96.7 (8.4)	57.1 (7.1)	92.1 (10.1)	106.9 (52.3)	72.2 (11.2)
CA	399 (41.3)	365 (42.2)	34 (33.3)	96.3 (8.0)	56.2 (6.8)	91.5 (9.0)	109.6 (50.2)	72.4 (11.6)
AA	88 (9.1)	78 (9.0)	10 (9.8)	95.1 (8.7)	56.6 (7.1)	91.5 (9.8)	118.5 (60.1)	75.0 (11.3)*
*rs4813627* GG (wild type)	277 (28.7)	247 (28.6)	30 (29.4)	97.2 (8.6)	57.1 (7.5)	92.0 (10.5)	107.3 (54.3)	72.0 (11.1)
GA	458 (47.4)	410 (47.5)	48 (47.1)	96.0 (7.8)	56.6 (6.8)	92.0 (9.4)	107.6 (51.2)	72.4 (11.5)
AA	231 (23.9)	207 (24.0)	24 (23.5)	96.1 (8.8)	56.6 (6.7)	91.3 (9.0)	114.1 (51.7)	73.4 (11.5)

SBP = Systolic blood pressure; DBP = diastolic blood pressure; HR = Heart rate; RSA = Respiratory Sinus Arrhythmia; PEP = Pre-ejection Period.

*<0.05

**<0.01

***<0.001

### Child’s *OXTR*, *OXT* genotype variants and cardiovascular outcomes at age 5–6

Genotype variant *rs2268498 TC* was associated with an increase in SBP of 1.9 mmHg (F = 8.819; *p* = 0.003) and in HR of 2.3 bpm (F = 8.529; *p* = 0.004) compared to the homozygote *rs2268498 TT* (wild type) variant. Genotype variant *rs2740210 AA* was associated with a larger pre-ejection period (PEP) of 2.8 msec (F = 4.644; *p* = 0.032) compared to the *rs2740210 CC* (wild type) variant. Genotype variants *rs53576* and *rs4813627* were not associated with cardiovascular outcomes (Table 2).

### Maternal verbally aggressive behavior in infancy and child’s cardiovascular outcomes at age 5–6

Maternal verbally aggressive behavior in infancy was associated with a higher SBP after adjustment for sex, height and age (Model 1) (B = 2.1 mmHg, 95% CI [0.4;3.8]) at age 5–6. Adjustment for potential confounding factors (Model 2) only slightly attenuated the association (B = 2.0 mmHg, 95% CI [0.2; 3.8]). Maternal verbally aggressive behavior in infancy was not associated with diastolic blood pressure (DBP), HR, PEP or respiratory sinus arrhythmia (RSA) at age 5–6.

### Child’s *OXTR* and *OXT* genotype variants, maternal verbally aggressive behavior in infancy and cardiovascular outcomes at age 5–6

**Fig 2:**
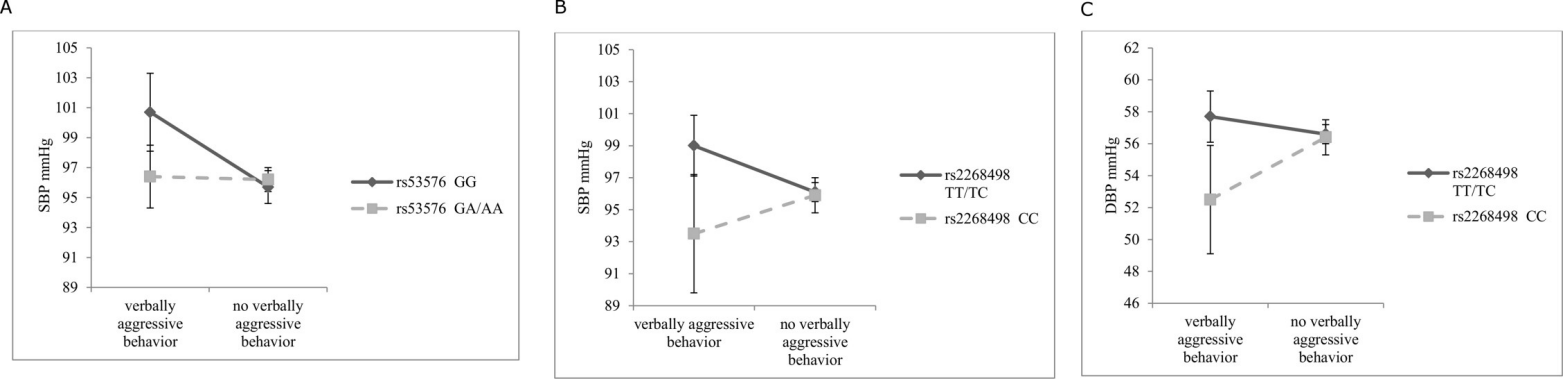
**A.** Systolic blood pressure according to maternal verbally aggressive behavior, stratified by *rs53576*. **B.** Systolic blood pressure according to maternal verbally aggressive behavior, stratified by *rs2268498*. **C.** Diastolic blood pressure according to maternal verbally aggressive behavior, stratified by *rs2268498*.

In line with our hypotheses, some interactions were shown to be significant, indicating that the association between maternal verbally aggressive behavior and blood pressure was dependent on the genotype. First, carriers of *rs53576GG* having experienced maternal verbally aggressive behavior in infancy, showed an increased SBP of 4.9 mmHg (95% CI 2.2; 7.7) at the age of 5–6 (Fig 2A), compared to unexposed carriers of *rs53576 GG* (*p*-value for interaction = 0.012). Secondly, carriers of *rs2268498 TT/TC* having experienced maternal verbally aggressive behavior in infancy, showed an increased SBP of 3.0 mmHg (95% CI 1.0; 5.0) at the age of 5–6 (Fig 2B), compared to unexposed carriers of *rs2268498 TT/TC* (*p*-value for interaction = 0.021). Carriers of *rs2268498 CC* having experienced maternal verbally aggressive behavior in infancy showed a decrease in DBP of -3.9 mmHg (CI -7.4;-0.4), compared to unexposed carriers of *rs2268498 CC* (*p-*value for interaction = 0.021) at the age of 5–6 (Fig 2C). Other interactions were not statistically significant (S1 Table).

## Discussion

Our study shows novel evidence suggesting that *OXTR* gene variants might influence the vulnerability to develop increased SBP at age 5–6, after exposure to maternal verbally aggressive behavior in early infancy. Confirming our hypothesis, we found evidence for gene- environment interaction of *OXTR* polymorphisms *rs53576* and *rs2268498*, both of which have previously been associated with facial emotion recognition [12,14]. Our findings of *rs53576* are in line with a retrospective study on childhood maltreatment and adult emotional dysregulation, in which carriers of *rs53576 GG* were at risk for increased emotional dysregulation when exposed to three or more categories of childhood abuse. On the contrary, A-allele carriers were suggested to be resilient against the effects of severe childhood adversity [22]. In our study, exposed A-allele carriers also seemed resilient to increases in SBP. We found no significant gene-early environment interaction with respect to the *OXT* gene variants *rs2740210* and *rs4813627*, and maternal verbally aggressive behavior in infancy in increasing SBP at age 5–6, although the non-significant interactions were in the same direction (S2A Fig and S2B Fig).

In contrast to our hypothesis, we found neither evidence for interaction of *OXTR* nor *OXT* gene variants with exposure to maternal verbally aggressive behavior in early infancy in increasing DBP, HR, or altering resting ANS activity at the age of 5–6. Associations might only start showing after exposure to multiple early life stressors, comparable to the study of Pretty et al. [23], in which four or more adverse childhood experiences were positively associated with HR. In line with this study, the combination of adverse life events and chronic stress has been shown to be associated with altered cardiac ANS activity, measured by heart rate variability (HRV) [24]. Another explanation for our null findings could be that we measured the children in rest. Differences in HR and ANS activity are much more likely to show when the system is stressed. In interpretation of null findings on DBP, it is important to consider that BP reactivity through an increase in adrenergic cardiac contractility will be measurable in SBP and not in DBP. Importantly, in children, SBP reactivity but not DBP or HR reactivity has been shown to predict carotid intima–media thickness, an index of diffuse subclinical atherosclerosis [25].

An unexpected finding was a decrease in DBP of -3.9 mmHg in carriers of *OXTR* variant *rs2268498 CC* (homozygote recessive type/ non risk allele) having experienced maternal verbally aggressive behavior in infancy. Although the exposed risk allele carriers display increased DBP, as compared to the exposed non-risk allele carriers, the DBP of the exposed risk allele carriers is comparable to the DBP of the non-exposed infants (and therefore not increased in absolute terms). Nonetheless, our findings could be interpreted being in the direction of the hypothesis: within infants exposed to maternal verbal aggression, those with risk genotype *rs2268498 TT/TC* display increased DBP as compared to non-risk-carriers *rs2268498 CC*.

As yet, no literature exists on possible differences in BP, HR and ANS activity directly due to presence or absence of *OXT* and *OXTR* polymorphisms. In our study, some genotype variants showed direct effects on cardiovascular outcomes. Replication is needed of both the direct effect of *rs2268498* on SBP and HR and the direct effect of *rs2740210* on PEP in order to further study the function of these SNPs in cardiovascular regulation.

Our finding of *OXTR* variant *rs2268498* related to vulnerability to increases in SBP after a context of early life stress is highly novel. Our results suggest that an infant’s genetic requisites of better facial emotion recognition, and higher social sensitivity, increases the vulnerability of the exposed child to an increase in SBP at age 5–6. Interestingly, from an evolutionary perspective, *OXTR* variants *rs53576 GG* (the wild type) and *rs2268498 TT/TC*, being more sensitive to the social environment, might create more cardiac output to support a necessary fight or flight response, an adequate environmental adaptation. However, an increased SBP in the long term carries a cardiovascular health risk. Since BP has been shown to track into adulthood, with tracking coefficients larger for SBP than DBP [26], the demonstrated increases of 3.0–4.9 mmHg in SBP at age 5–6 in the normal population can be considered large and clinically relevant in the future. Moreover, elevated BP in children has been shown to be associated with hypertensive target-organ damage [27].

### Strengths and limitations

Strengths of this study are the large population-based birth cohort, the prospective study design, the extensive data collection from early infancy onwards and BP, HR and ANS measurements according to a standardized protocol. Importantly, we were able to control for a large number of potentially confounding stressors, such as maternal physical aggressive behavior in infancy and authoritarian parenting style at age 5–6. We specifically excluded participants with previously known physiological variables of influence on the ANS and BP such as preterm birth, which is associated with increased sympatho-adrenal activity in childhood [28].

Based on literature on sample size, statistics and selection of SNPs in genetic epidemiologic studies [29–32], a maximum of four SNPs were selected for investigation. These four SNPs have previously been associated with either recognition of facial emotions or vocalizing and have a MAF> 0.25. As maternal verbally aggressive behavior did not differ between responders and non-responders (those who did not participate at age 5–6), respectively 10.6% and 9.2% (p = 0.177), possible selection bias is estimated to be limited. Importantly, the genotype variants did not vary across the exposed and non-exposed infants. Therefore no population stratification was present. Also, time of day of measurements did not differ between the genotypes.

Maternal verbally aggressive behavior was considered present if speaking angrily had been present twice or more, at the infant’s age of 3 months. This definition of maternal verbally aggressive behavior seems quite narrow, but, at the same time, encloses all from only twice to frequent exposure to maternal verbally aggressive behavior in early infancy. Face validity of maternal self-reported verbally aggressive behavior will depend on maternal self-reflection. In our study, we did not have information on maternal self-reflection. Therefore, we could not include this covariate in our analyses. Importantly, self-reflection can be influenced by depressive symptoms in a way that it could lead to overestimation of behavior. However, we do not expect mothers to overestimate verbally aggressive behavior. On the contrary, possible social desirability could lead to underestimation of the true frequency of maternal verbally aggressive behavior. Importantly, self-report is the only way to measure verbally aggressive behavior, as continuous observation of the mother-infant dyad is not feasible. In our analyses we did adjust for maternal depressive symptoms both in infancy and at age 5–6. A limitation is that the distinction between temporary, frequent or persistent maternal verbally aggressive behavior to the infant, thus creating chronic stress in the first years, could not be made based on our data. Due to small numbers we were not able to investigate a possible dose-response relationship. Also, we could not assess the possible contribution of paternal aggressive behavior. We suspect that experience of simultaneous other adverse events will probably increase the gene-environment interaction effect.

Although the study population was large, the numbers of subjects in various groups in the studied interactions was fairly small (40 exposed children with *OXTR rs53576 GG* (wild type); 19 exposed children with *OXTR rs2268498 CC* (homozygote recessive type); 44 exposed children *OXT rs2740210 CC* (wild type); 30 exposed children with *OXT rs4813627 GG* (wild type)). In order to follow the recommendations of Keller (2014) to properly control for potential confounders [33] we additionally checked whether adding sex*G and sex*E to the complete model would change the results. The *p*-values for interaction of the SNPs and maternal verbal aggression in association to SBP minimally changed, and remained significant (rs53576* verbal aggression: *p* = 0.016 (instead of 0.012); rs2268498* verbal aggression: *p* = 0.026 (instead of 0.021). We aimed to replicate the results in an independent cohort, but, as far as we know, no other cohort study exists in which both maternal verbally aggressive behaviour at the age of 3 months and blood pressure at age 5 were assessed. Nevertheless, this is an exploratory analysis and our findings on gene-environment interaction by *OXTR* polymorphisms should be replicated in independent samples.

Furthermore, gene-environment interaction of *OXTR* or *OXT* polymorphisms could differ between boys and girls. Indeed, evidence of sex differences exists in brain structure, function, and neurotransmission [34] as well as stress-reactivity [35]. Unfortunately, our study was underpowered for sex-stratified interaction analyses. Of note, it is unknown whether the selected SNPs in our study have similar or overlapping functions and the small numbers hindered investigation of gene-gene interactions.

### Potential underlying mechanisms

Exposed carriers of risk alleles enhancing social sensitivity are suggested to be vulnerable to an increase in SBP at age 5–6. These infants might have experienced higher stress levels than non-risk allele carriers. These physiological stress levels could have induced programming of biobehavioral systems by affecting the developing brain, ultimately impacting the child’s SBP at age 5–6.

A first mechanism explaining our findings could be a direct effect of the gene- environment interaction on programming of OXT levels. In a retrospective study, multiple stressful early life events have been linked to reduced OXT levels in the Cerebro Spinal Fluid (CSF) [36], suggesting a programming effect which in itself might increase SBP. Although the functional effects of *OXTR* or *OXT* polymorphisms on receptor binding and OXT levels are not known, *OXTR* polymorphisms might affect programming of density and regional distribution of the *OXTR* by epigenetic mechanisms, and thereby differentially affect OXT levels and consequently oxytocinergic cardioprotection. Importantly, early life experiences might alter sensitivity to both oxytocin and vasopressin systems [37] through epigenetic modification. Notably, *OXTR* is coupled to Gq, activating the same intracellular pathways as arginine-vasopressin receptor *(AVPR)1a* and *AVPR1b* [38]. Some evidence exists on the formation of heterodimers between these three receptors [39], which all three are simulated by vasopressin [40] and potentially impact BP.

Secondly, striatal volume may be differentially affected by exposure to stress in early life depending on whether the child is a risk-carrier or not. The striatum consists of the ventral striatum (nucleus accumbens and olfactory tubercle) and dorsal striatum (caudate nucleus and putamen) and is activated by unexpected or intense stimuli [41]. Between 2–4 months, synaptogenesis in the striatum is most rapid and total gray matter volume of the striatum reaches adult size at about age 4 months already [1]. Stress during this early period might have distinct programming effects, which could impact cardiovascular outcomes in children. Interestingly, in the context of childhood maltreatment, *rs53576 GG* homozygotes, carriers of risk alleles enhancing social sensitivity, display a strong gray matter reduction of bilateral ventral striatum with increasing Childhood Trauma Questionnaire (CTQ) scores as adults [12], implying programming effects. It is plausible that a gray matter reduction of the ventral striatum will affect its functionality. Indeed, a decrease of reward dependence has been found present more often in *G-*allele carriers, being socially sensitive, who had been exposed to early life adverse events [12].

Finally, programming of striatal systems, such as the renin-angiotensin system (RAS) and dopaminergic system could differ between the risk and the non-risk carriers. Major components of the RAS, a BP regulator, have been shown to exist in striatal astrocytes and microglial cells [42]. Hyperactivation of RAS has been shown to exacerbate oxidative stress and microglial inflammatory response and to contribute to progression of dopaminergic degeneration in animal models. A decrease in dopaminergic activity has been shown to induce compensatory upregulation of local RAS function in both dopaminergic neurons and glia cells [43], hereby accelerating hyperactivation. Increased stress reactivity in exposed carriers of risk alleles enhancing social sensitivity may induce local RAS hyperactivation. Ultimately, SBP might be increased by these programming effects. Moreover, local dopaminergic activity might be altered. Interestingly, after exposure to early life stress (induced by repeated parental separation) a programming effect on increased striatal Dopamine Active Transporter (DAT) availability has been shown in rats [44]. In line with the evidence that increased striatal DAT availability precedes hypertension in rats [45], a possible increase in striatal DAT availability due to programming effects might impact BP in the child at age 5–6 as well. Of course, early neglect in rats is not fully comparable to the stressor of maternal verbally aggressive behavior in infants.

Essentially, in *rs53576 G-*carriers only, peripheral oxytocin level has been negatively correlated with striatal DAT availability [46]. This specific difference between *rs53576* variants might be causing an additional difference between carriers and non-carriers of the risk allele. Although speculative, *G-*carriers might not be able to lower an increased SBP due to programming effects in several systems.

Concluding, maternal verbally aggressive behavior may be an important early life stressor with negative impact on cardiovascular health later in life. Infant’s variation of *OXTR* related genes are suggested to be a significant factor of vulnerability to increases in SBP at age 5–6. Future studies may address whether the GxE interactions we demonstrated, can be replicated in larger birth cohort studies, in adolescence and adulthood, whether differences between boys and girls exist and whether other cardiovascular outcomes are affected when stress is induced. Finally, studies of possible genetic overlapping functions are needed to further explore the relevance of these promising genetic markers.

## Methods

### Participants

The study sample is part of a large prospective, observational, population-based multiethnic birth cohort, the Amsterdam Born Children and their Development (ABCD) study, which started in 2003. Extensive information about the cohort and procedures regarding data collection has been published elsewhere [47]. Data were processed anonymously. Approval of the ABCD study was obtained from local ethical committee (Institution name Academic Medical Hospital, approval number MEC 02/39#02.17.392). Approval of the ABCD-Genetic Enrichment (ABCD-GE) study, a sub-study of 1192 Dutch children, was obtained from the Medical Ethical Committees of the Academic Medical Hospital in Amsterdam. For the ABCD-GE study an opt out procedure was followed. All participating mothers gave written informed consent for themselves and their children.

**Fig 3:**
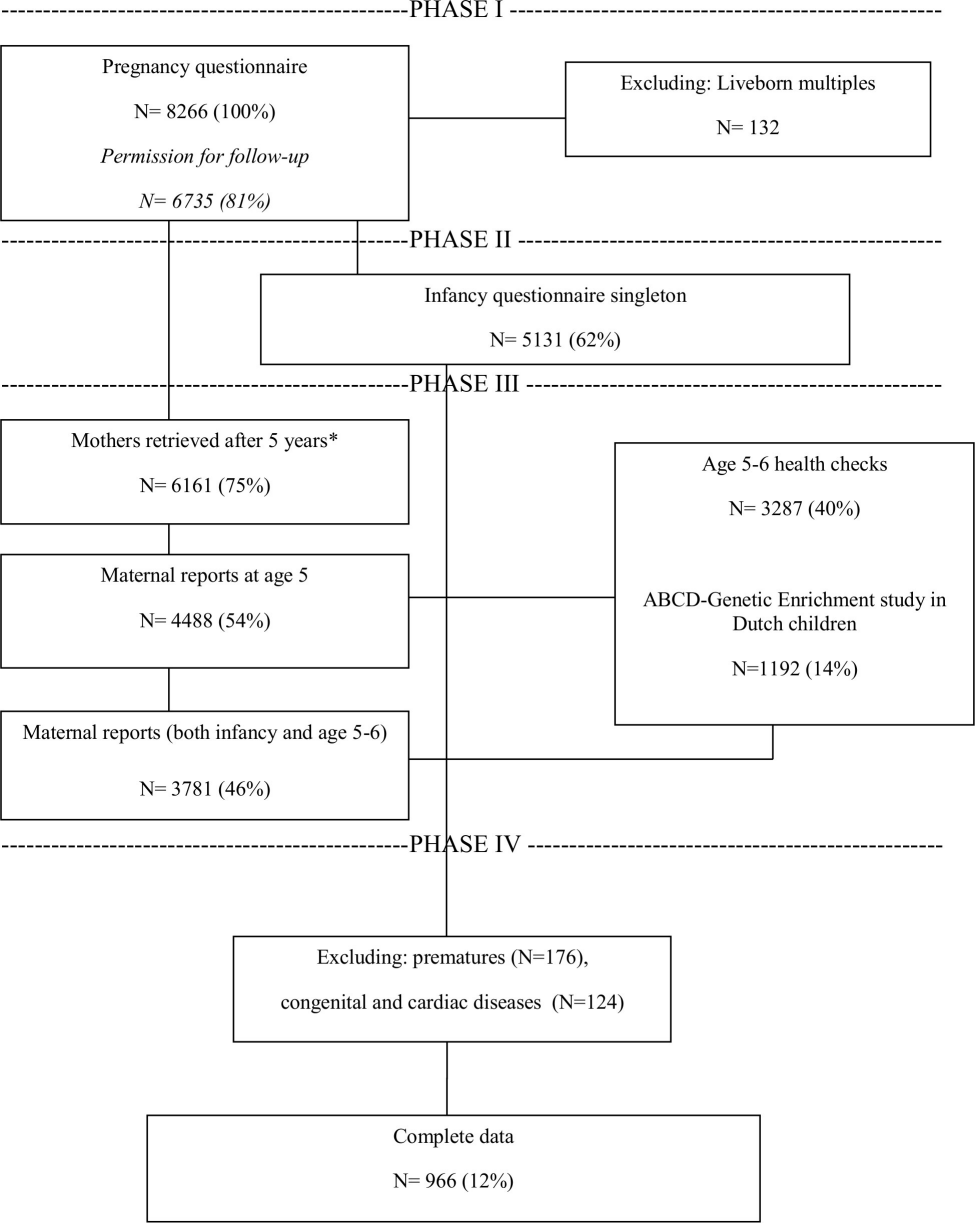
Flow chart of participants included for analysis.

Between January 2003 and March 2004, all pregnant women living in Amsterdam were asked to participate in the ABCD study during their first prenatal visit to an obstetric care provider (general practitioner, midwife, or gynecologist). Of the 12373 women approached, 8266 women filled out the pregnancy questionnaire (mean gestational age: 15.7 weeks (SD 3.5) (response rate: 67%)). A total of 6735 women gave permission for follow-up. Three months after birth, 5131 women filled out the infancy questionnaire. For the questionnaire at the child’s age of 5–6, addresses of 6161 mothers were retrieved; 4488 mothers returned the questionnaire. Attrition in this follow-up number was due to withdrawal, unknown address, infant or maternal death. Multiple births were excluded from the cohort preceding the third phase. The health check at 5–6 years consisted of various health measurements in 3287 children aged 5–6 years (2008–2010; mean age 5.7 years +- 0.5 SD). For the ABCD-Genetic Enrichment (ABCD-GE) study, blood of the child was collected from a simple finger prick during the 5-year health check-up in 1192 children of Dutch descent. DNA was extracted from the dried blood spots. Data on all three measurements (pregnancy, infancy, and early childhood) had to be available to be included in the present study. We excluded preterm births (gestational age < 37 weeks) and congenital diseases (N = 966 included). The flow chart of participants included in the present study is shown in Fig 3.

### Maternal verbally aggressive behavior in infancy

Maternal verbally aggressive behavior was assessed by a maternal self-report questionnaire completed in the 13^th^ week after birth (range 11–25 weeks, SD 2 weeks). Maternal verbally aggressive behavior was assessed using a question about the frequency of speaking angrily to the infant on a six-point scale: ‘Have you ever spoken angrily to your baby in order to diminish the crying?’ The histogram of the answers to this question is shown in S1 Fig. The score was dichotomized [speaking angrily (frequency < = 1 or > = 2)]. Maternal verbally aggressive behavior was considered present if speaking angrily to the infant had been present twice or more. This cut-off was chosen in order to make a distinction between speaking angrily to the infant by accident, versus twice or more often [3].

### Child cardiac autonomic nervous system activity, blood pressure, and heart rate at age 5–6

Cardiac ANS activity was assessed at the age 5 health check, using the VU University Ambulatory Monitoring System (VU-AMS, version 5fs, TD-FPP, Amsterdam, the Netherlands). Reliability and validity aspects and recording methodology of the VU-AMS have been described previously [48]. The system records three lead electro cardiograms (ECG) and four lead impedance cardiograms (ICG) (Ultratrace Diagnostic ECG with wet gel; ConMed Corporation, Utica, New York, United States of America). In brief, ECG and respiratory activity were recorded between 8:30 am and 15:00 pm in sitting and supine positions, during which no conversation took place. RR-interval time series were analyzed during 4 minutes. Mean heart rate (HR) was 85.5 bpm (60 sec/85.5 = 0.7 sec for RR-interval). All R-peaks in the ECG were checked and R-peak markers were moved, inserted or deleted. The software automatically marked inspirations and expirations in the respiratory signals, which were manually checked. No edits were necessary [48].

Respiratory Sinus Arrhythmia (RSA), a time domain index of Heart Rate Variability (HRV) in the respiratory frequency range, was automatically obtained as derivate of parasympathetic nervous system activity. RSA was taken as the peak valley estimation (pvRSA) which was obtained automatically by subtracting the shortest inter beat interval during HR acceleration in the inspirational phase from the longest inter beat interval during deceleration in the expirational phase. Decreased parasympathetic activity is indicated by a shorter RSA. Pre-ejection period (PEP) is a measure of sympathetic activity. PEP can reliably be measured in children [49]. PEP is the time interval between the onset of the ventricular depolarization (Q wave onset in the ECG) and the time of opening of the aortic valves (B point in the ICG) and was scored manually in large-scale ensemble averages of the impedance cardiograms. Decreased sympathetic activity is indicated by a longer PEP [50]. To obtain the mean value of the HR, the mean HR across the experimental conditions was used, excluding the period during which BP was measured.

Children’s BP was measured twice both in supine and sitting position after a test reading and 5 minutes of rest. BP was measured with the automatic oscillometric method, using the Omron 705 IT (Omron Healthcare Inc., Cannockburn, IL, USA) with a small cuff (arm circumference 17–22 cm) on the non-dominant arm. When either the systolic (SBP) or diastolic pressure (DBP) differed more than 10 mmHg between the two measurements, a third measurement was taken (17%). The average of the two measurements closest together was used in the supine and sitting position. For this study we used PEP, RSA, HR and BP in sitting position.

### Genotyping

The finger prick yielded fasting capillary blood samples for DNA extraction. Subsequently, the DNA samples were genotyped, using the Illumina Human Core Exom Beadchip (Illumina, San Diego, CA, USA). The Illumina Human Core Exom Beadchip includes over 540,000 genetic markers. Before imputation, SNPs were excluded if they had high levels of missing data (SNP call rate < 95%, strong departures from Hardy-Weinberg equilibrium (HWE) (P<1 x 10^−^*6*), or low minor allele frequencies (MAF) (<1%). Moreover, if mismatch in heterozygosity, gender or relatedness existed, individuals were excluded. Genetic markers were imputed using the IMPUTE2 software and the 1000 Genomes References Panel (phase 1 release v3, build 37). Genotypes for the SNPs of interest were extracted from the imputed GWAS data set. The final number of SNPs was 277,644. The total of SNPs after imputation was 27,448,454. The mean quality of the imputed genotypes (r^2^) of the 4 SNPs included in this study (*rs53576*, *rs2268498*, *rs2740210*, and *rs4813627*) was 0.82, ranging from 0.75 to 0.87 (Table 3). Using PLINK, none of the selected SNPs were in pair-wise linkage disequilibrium (R^2^ = 0.31 and 0.28 for *rs53576/rs2268498* and *rs2740210/rs4813627*, respectively).

**Table 3:** Oxytocin polymorphism variants and imputation quality.

Chr	rs_id	Bp position	Minor/ Major allele	MAF	Coded allele	Quality of imputation	Certainty	HWE P-value
3	*rs53576*	8804371	A/G	0.35	G	0.87	0.925	0.52
3	*rs2268498*	8812411	C/T	0.45	C	0.87	0.916	0.91
20	*rs2740210*	3053255	A/C	0.30	A	0.75	0.866	0.73
20	*rs4813627*	3055513	A/G	0.50	G	0.77	0.851	0.19

MAF = Minor Allele Frequency; HWE = Hardy-Weinberg equilibrium

### Covariates

The following child characteristics during infancy were included from youth health care services: sex, birth weight, and gestational age. Excessive infant crying was assessed by questionnaire. Excessive infant crying was considered present if mothers estimated their infant to cry for three or more hours per 24 hour day on average in the 13^th^ week after birth (range 11–25 weeks, SD 2 weeks) (best approximation of the Wessel’s criteria) [51].

The following maternal characteristics were included during pregnancy: age, parity (0 or > = 1), cohabitation status (single or living together with partner), and level of education (years after primary school). During infancy, maternal depressive symptoms, maternal physically aggressive behavior and maternal smoking at home were included. Maternal postnatal depressive symptoms were measured using the Center for Epidemiologic Studies Depression Scale (CES-D) [52]. The summed scores were analyzed continuously. Pleasure in infant care was measured using five questions on a four point scale (very true, true, not true, not true at all): ‘I feel very good taking care of my baby?’; ‘I feel very satisfied taking care of my baby?’; ‘I feel happy taking care of my baby?’; ‘I am fed up taking care of my baby?’; ‘I really enjoy taking care of my baby?’ The summed scores were analyzed continuously. Maternal physically aggressive behavior consisted of three behaviors on a six-point scale, which were dichotomized [cloth on mouth (frequency 0 or > = 1), slapping (frequency 0 or > = 1), and shaking baby (frequency 0 or > = 1)]. These three behaviors were summed scored as maternal physically aggressive behavior, and analyzed continuously. Maternal smoking at home during infancy was either present or absent (yes or no).

Additionally the following characteristics were included: maternal pre-pregnancy BMI (kg/m2) and preexistent hypertension (yes or no). Pregnancy-induced hypertension (yes or no) was available by combining data from the questionnaire and the Dutch Perinatal Registration (PRN, www.perinatreg.nl) and classified in accordance with the guidelines of the International Society for the Study of Hypertension in Pregnancy (www.isshp.com). Paternal hypertension and family (maternal or paternal) hypertension were derived from a questionnaire previously described elsewhere [53].

Covariates at age 5 included the exact age of the child at the 5–6 years health check and time of day when measurements were taken. The height and weight of the child were measured from which BMI was calculated. Authoritarian parenting style was measured by the 12-items subscale of the short version of the Parenting Styles and Dimensions Questionnaire (PSDQ) [54] and analyzed continuously. Maternal parenting stress was measured by nine items on attachment derived from the 123-items of the ‘Nijmeegse Ouderlijke Stress Index’ (NOSI-K) [55] and was analyzed continuously. Maternal depressive symptoms at age 5 were measured by the depression severity subscale of the Depression Anxiety Stress Scales (DASS 21) [56] and were analyzed continuously. Whether the child was being raised in an intact family (yes or no) was assessed by one item from the questionnaire filled out at age 5–6.

### Statistical analyses

All variables were inspected for outliers and checked on normality. Descriptive statistics were used to describe maternal and child characteristics. Associations between these characteristics and maternal verbally aggressive behavior were tested using analysis of variance for continuous variables and Chi square tests for categorical variables. Associations between the different polymorphisms and maternal verbally aggressive behavior, SBP, DBP, HR, RSA and PEP were tested using analysis of variance for continuous variables and Chi square tests for categorical variables. The associations of maternal verbally aggressive behavior and ANS, BP and HR were analyzed by means of multivariable linear regression analysis. Potential confounders were selected a priori from Table 2 and included in the regression model by using a forced- entry method. We selected covariates associated with maternal verbally aggressive behavior as possible confounders. After initial testing in a crude univariate model, or a minimally adjusted model for sex, age and height of the child (model 1), the following covariates were added to a fully adjusted model (model 2): postnatal depressive symptoms, pleasure in infant care, maternal physically aggressive behavior in infancy, authoritarian parenting style, maternal depression and parenting stress (NOSI-K) at the child age of 5–6. Interaction terms of maternal verbally aggressive behavior and polymorphism variant of OXTR *rs53576* (*GG* vs. *GA/AA*) or *rs2268498* (*TT/TC* vs. *CC*) and maternal verbally aggressive behavior and OXT *rs2740210* (*CC* vs. *CA/AA*) or *rs4813627* (*GG* vs *GA/AA*) were added to the fully adjusted models to test interaction in risk alleles. Additionally, the data were stratified into two groups (presence or absence of *OXTR* risk alleles according to the hypothesis or *OXT* wild type or not) and analyzed separately according to the above model. The statistical analyses were performed by IBM SPSS Statistics software version 20.0 (IBM Corp, Armonk, NY, USA). The significance level we used in the study was 5%.

## Supporting information

S1 Fig Frequency of speaking angrily. Frequency of speaking angrily = ‘Have you ever spoken angrily to your baby in order to diminish the crying?’ (1 = no; 2 = 1 time; 3 = 2 times; 4 = 3 times; 5 = 4 times; 6 = 5 times or more). Maternal verbally aggressive behavior was considered present if speaking angrily to the infant had been present twice or more.
(TIF)

S2 Fig (a) Systolic blood pressure (mmHg) according to maternal verbally aggressive behavior, stratified by *rs2740210*. Carriers of *rs2740210 CA/AA* exposed to maternal verbally aggressive behavior in early infancy have a higher SBP of 3.9 mmHg (95%CI 1.2;6.6) than unexposed carriers of *rs2740210 CA/AA* (p-value for interaction = 0.135). (b) Systolic blood pressure (mmHg) according to maternal verbally aggressive behavior, stratified by *rs4813627*. Carriers of *rs4813627 GA/AA* exposed to maternal verbally aggressive behavior in early infancy have a higher SBP of 2.8 mmHg (95%CI 0.7;4.9) than unexposed carriers of *rs4813627 GA/AA* (*p*-value for interaction = 0.193).
(ZIP)

S1 Table Differences in heart rate, blood pressure and resting ANS at age 5–6 years between children exposed to maternal verbally aggressive behavior in infancy and non-exposed children, stratified for presence or absence of oxytocin genetic risk variants. Model 1: adjusted for sex, height and age of the child; Model 2: Model 1, additionally adjusted for maternal depressive symptoms, pleasure in infant care, maternal physical aggression in infancy, authoritarian parenting style, maternal depressive symptoms and parenting stress (NOSI-K) at the child’s age of 5–6. *Interaction between maternal verbal aggressive behavior in infancy and oxytocin polymorphism *<0.05; **<0.01; ***<0.001
(PDF)
