# Supplementary figures and images for: Common oxytocin polymorphisms interact with maternal verbal aggression in early infancy impacting blood pressure at age 5-6: The ABCD study
Source: PLoS One. 2019 Jun 24;14(6):e0216035. doi: 10.1371/journal.pone.0216035 (PMC6590781; doi:10.1371/journal.pone.0216035)

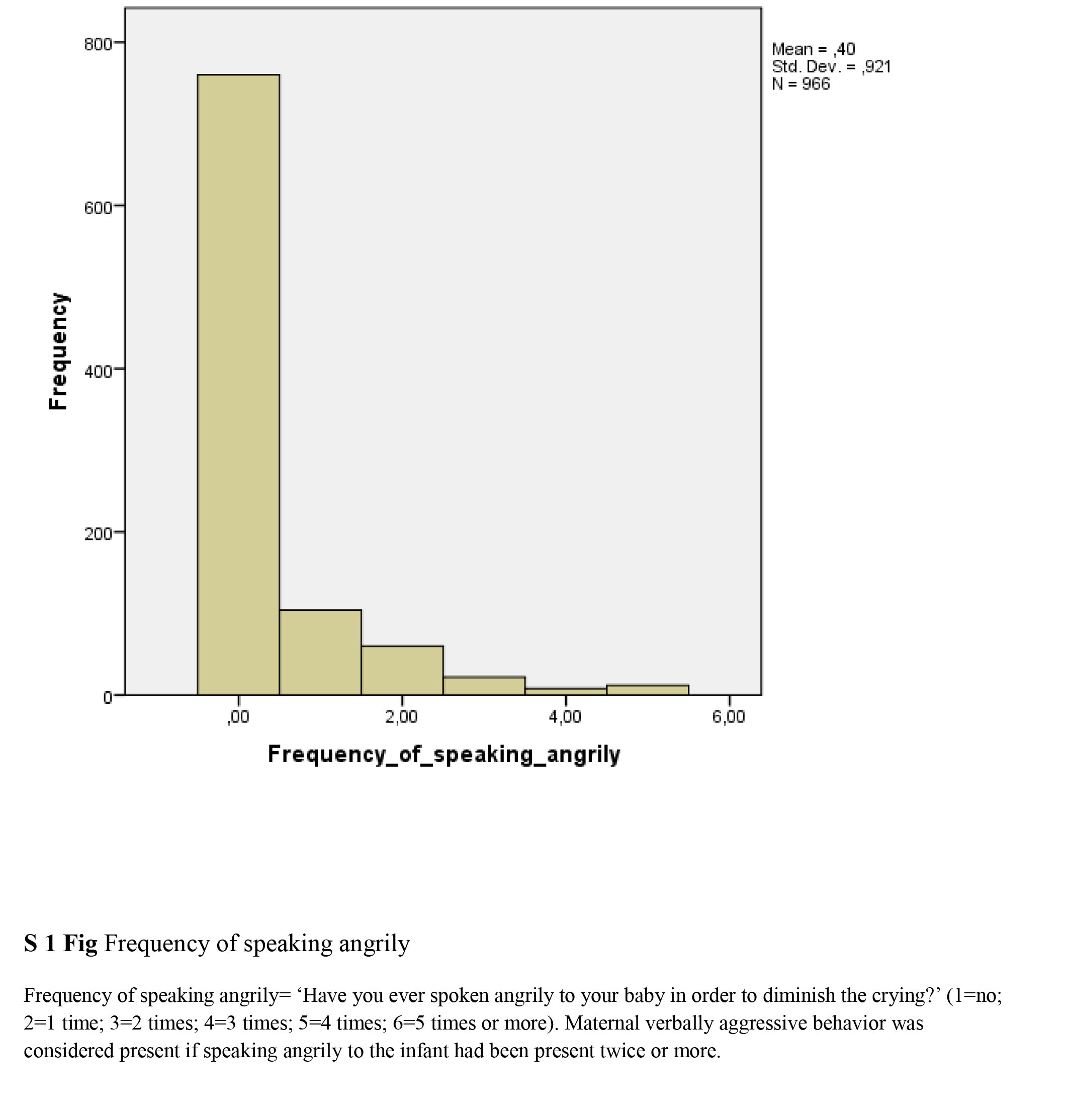

Supplement: S1 Fig — Frequency of speaking angrily = ‘Have you ever spoken angrily to your baby in order to diminish the crying?’ (1 = no; 2 = 1 time; 3 = 2 times; 4 = 3 times; 5 = 4 times; 6 = 5 times or more). Maternal verbally aggressive behavior was considered present if speaking angrily to the infant had been present twice or more. (TIF) [file pone.0216035.s001.tif]

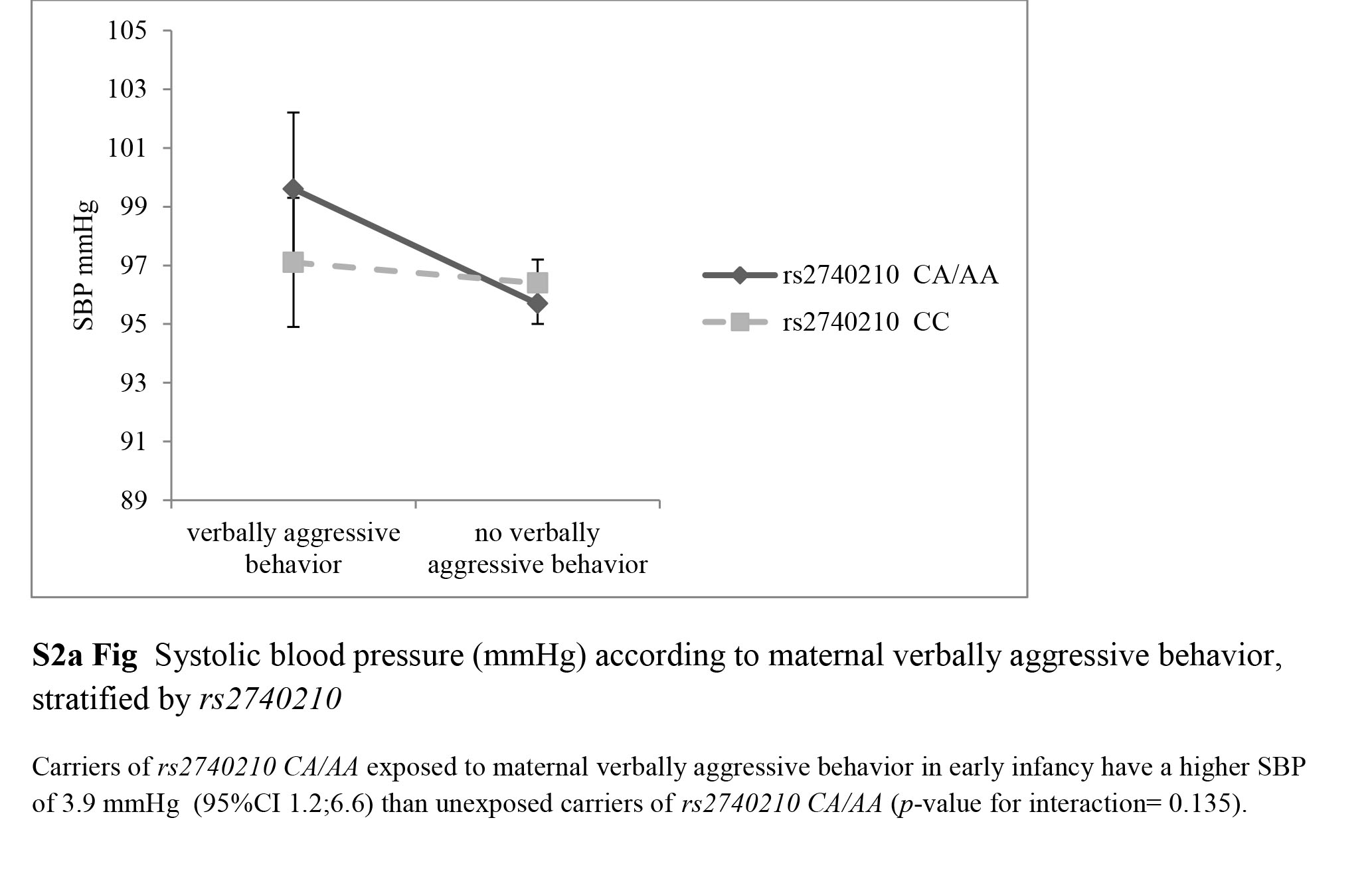

Supplement: S2 Fig — (a) Systolic blood pressure (mmHg) according to maternal verbally aggressive behavior, stratified by rs2740210. Carriers of rs2740210 CA/AA exposed to maternal verbally aggressive behavior in early infancy have a higher SBP of 3.9 mmHg (95%CI 1.2;6.6) than unexposed carriers of rs2740210 CA/AA (p-value for interaction = 0.135). (b) Systolic blood pressure (mmHg) according to maternal verbally aggressive behavior, stratified by rs4813627. Carriers of rs4813627 GA/AA exposed to maternal verbally aggressive behavior in early infancy have a higher SBP of 2.8 mmHg (95%CI 0.7;4.9) than unexposed carriers of rs4813627 GA/AA (p-value for interaction = 0.193). (ZIP) [file pone.0216035.s002.zip › S2a Fig.tif]

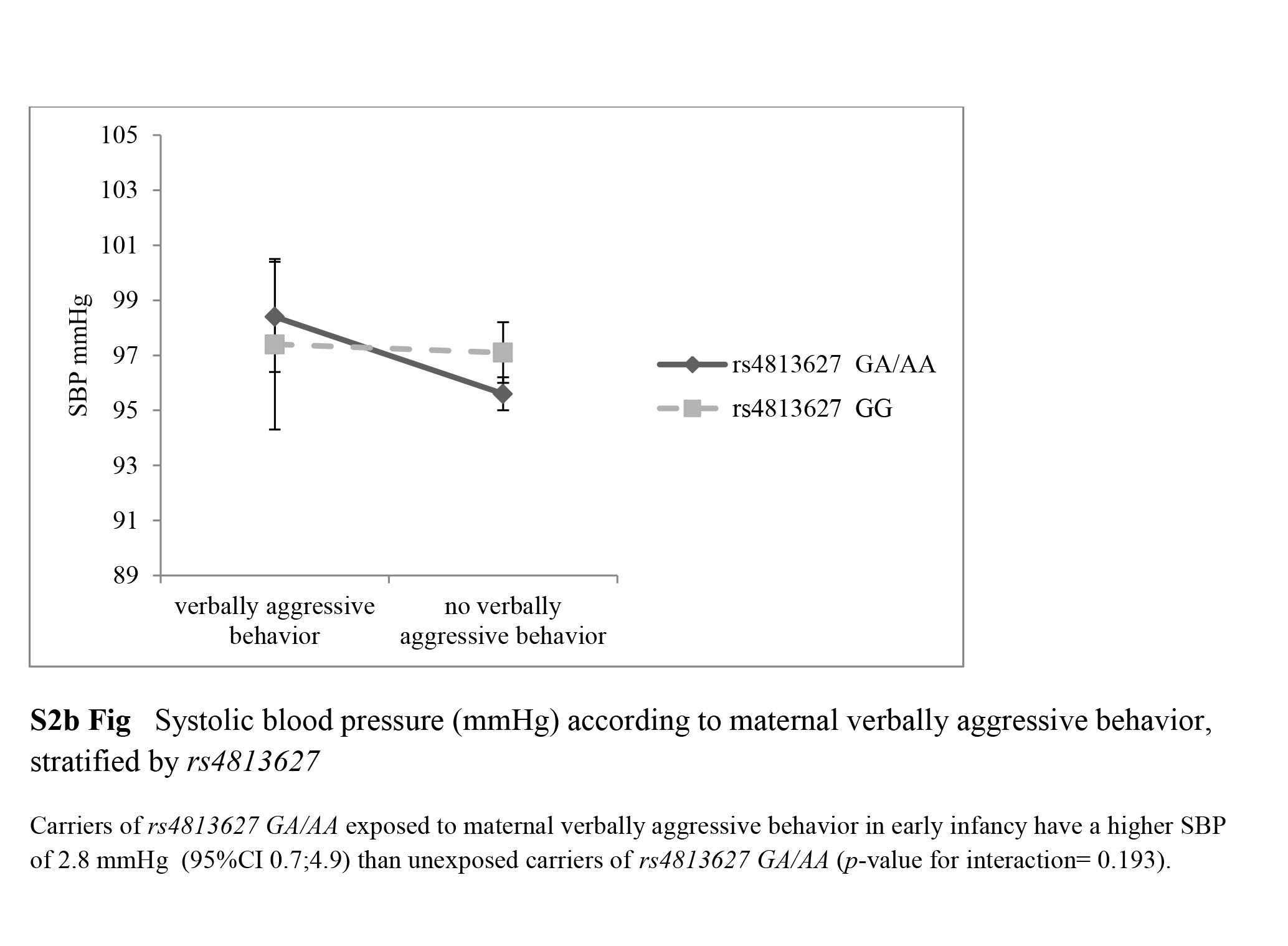

Supplement: S2 Fig — (a) Systolic blood pressure (mmHg) according to maternal verbally aggressive behavior, stratified by rs2740210. Carriers of rs2740210 CA/AA exposed to maternal verbally aggressive behavior in early infancy have a higher SBP of 3.9 mmHg (95%CI 1.2;6.6) than unexposed carriers of rs2740210 CA/AA (p-value for interaction = 0.135). (b) Systolic blood pressure (mmHg) according to maternal verbally aggressive behavior, stratified by rs4813627. Carriers of rs4813627 GA/AA exposed to maternal verbally aggressive behavior in early infancy have a higher SBP of 2.8 mmHg (95%CI 0.7;4.9) than unexposed carriers of rs4813627 GA/AA (p-value for interaction = 0.193). (ZIP) [file pone.0216035.s002.zip › S2b Fig.tif]
