# Supplementary material for: Common oxytocin polymorphisms interact with maternal verbal aggression in early infancy impacting blood pressure at age 5-6: The ABCD study
Source: PLoS One. 2019 Jun 24;14(6):e0216035. doi: 10.1371/journal.pone.0216035 (PMC6590781; doi:10.1371/journal.pone.0216035)
Supplement: S1 Table — Model 1: adjusted for sex, height and age of the child; Model 2: Model 1, additionally adjusted for maternal depressive symptoms, pleasure in infant care, maternal physical aggression in infancy, authoritarian parenting style, maternal depressive symptoms and parenting stress (NOSI-K) at the child’s age of 5–6. *Interaction between maternal verbal aggressive behavior in infancy and oxytocin polymorphism *<0.05; **<0.01; ***<0.001 (PDF) [file pone.0216035.s003.pdf]

S 1 Table

Difference in heart rate, blood pressure and resting ANS at age 5-6 years between children exposed to maternal verbally aggressive behavior in infancy and children not exposed to maternal verbally aggressive behavior in infancy, stratified by oxytocin polymorphism variants

	**Oxytocin Polymorphism** *rs53576*	Maternal verbally aggressive behavior in infancy No Mean (SD)	Yes Mean (SD)	Crude model Difference (95%CI)	Model 1 Adjusted difference (95%CI)	Model 2 Adjusted difference (95%CI)	Inter-action p-value*
Systolic BP (mmHg)	GG	95.7 ± 7.8	101.3 ± 9.9	5.60 (2.97;8.24)***	4.93 (2.30;7.55)**	4.94 (2.16;7.72)**	0.012
	GA/AA	96.4 ± 8.3	96.7 ± 8.7	0.32 (-1.89;2.53)	0.24 (-1.95;2.43)	0.12 (-2.22;2.46)	
Diastolic BP (mmHg)	GG	56.8 ± 7.3	58.4 ± 5.4	1.62 (-0.71;3.95)	1.80 (-0.53;4.14)	1.71 (-0.75;4.16)	0.208
	GA/AA	56.6 ± 6.8	56.3 ± 7.7	-0.36 (-2.18;1.46)	-0.56 (-2.42;1.30)	-0.76 (-2.75;1.24)	
Heart rate (bpm)	GG	91.7 ± 9.9	91.4 ± 9.3	-0.30 (-3.74;3.14)	0.39 (-3.03;3.80)	0.63 (-3.00;4.25)	0.938
	GA/AA	91.9 ± 9.5	91.8 ± 9.0	-0.13 (-2.79;2.53)	0.29 (-2.38;2.96)	0.33 (-2.53;3.18)	
RSA (msec)	GG	110.1±54.4	111.7±50.0	1.52 (-16.44;19.48)	1.71 (-16.46;19.89)	3.57 (-15.73;22.86)	0.646
	GA/AA	108.4±51.4	107.3±49.0	-1.04 (-14.92;12.84)	-2.53 (-16.73;11.68)	-2.48 (-17.59;12.62)	
PEP	GG	71.9±10.9	72.0±11.7	0.08 (-3.45;3.68)	-0.33 (-3.96;3.29)	-0.84 (-4.64;2.97)	0.820

(msec)	GA/AA	73.0±11.6	72.6±12.0	-0.41 (-3.58;2.75)	-1.37 (-4.55;1.82)	-0.77 (-4.18;2.65)	
	**Oxytocin Polymorphism *rs 2268498***	Maternal verbally aggressive behavior in infancy No Mean (SD)	Yes Mean (SD)	Crude model Difference (95%CI)	Model 1 Adjusted difference (95%CI)	Model 2 Adjusted difference (95%CI)	Inter-action p-value*
Systolic BP (mmHg)	TT/TC	96.1 ± 8.1	99.5 ± 9.9	3.38 (1.47;5.28)**	2.99 (1.10;4.78)**	2.96 (0.95;4.97)**	0.021
	CC	96.0 ± 7.9	94.2 ± 5.5	-1.82 (-5.52;1.78)	-2.02 (-5.77;1.73)	-2.41 (-6.39;1.57)	
Diastolic BP (mmHg)	TT/TC	56.8 ± 7.0	57.8 ± 7.2	0.99 (-0.62;2.61)	1.05 (-0.59;2.68)	1.06 (-0.68;2.80)	0.021
	CC	56.3 ± 6.7	54.2 ± 5.0	-2.18 (-5.30;0.95)	-3.27 (-6.55;0.02)	-3.90 (-7.42:-0.39)*	
Heart rate (bpm)	TT/TC	92.1 ± 9.7	91.4 ± 9.0	-0.73 (-3.08;1.61)	-0.25 (-2.59;2.10)	-0.01 (-2.50;2.49)	0.413
	CC	90.7 ± 9.4	92.8 ± 9.4	2.03 (-2.73;6.78)	2.29 (-2.63;7.21)	2.13 (-3.15;7.40)	
RSA (msec)	TT/TC	109.7±53.2	112.3±50.7	2.57 (-9.83;14.97)	2.15 (-10.41;14.71)	2.82 (-10.59;16.23)	0.252
	CC	106.7±50.5	95.6±40.6	-11.15 (-34.86;12.56)	-13.45 (-38.46;11.56)	-15.34 (-41.99;11.33)	
PEP (msec)	TT/TC	72.1±11.1	72.3±12.1	0.12 (-2.51;2.73)	-0.67 (-3.27;1.93)	-0.54 (-3.31;2.23)	0.773
	CC	74.0±12.0	72.7±10.9	-1.39 (-7.07;4.30)	-2.10 (-8.08;3.88)	-1.15 (-7.51;5.20)	
	**Oxytocin Polymor**	Maternal verbally aggressive behavior in infancy		Crude model	Model 1	Model 2	Inter-action

	**phism**<br><br>***rs 2740210***	No<br><br>Mean (SD)	Yes<br><br>Mean (SD)	Difference (95%CI)	Adjusted difference (95%CI)	Adjusted difference (95%CI)	p-value*
Systolic BP (mmHg)	CC	96.6 ±8.1	97.7 ±10.1	1.09 (-1.23;3.40)	0.93 (-1.36;3.22)	0.73 (-1.68;3.13)	0.135
	CA/AA	95.7 ±8.0	99.7 ±8.5	3.99 (1.48;6.49)**	3.60 (1.08;6.12)**	3.92 (1.21;6.62)**	
Diastolic BP (mmHg)	CC	57.2 ±7.0	56.5 ±7.7	-0.77 (-2.72;1.19)	-0.58 (-2.57;1.41)	-0.79 (-2.90;1.31)	0.267
	CA/AA	56.1 ±6.9	57.9 ±5.8	1.76 (-0.36;3.87)	1.25 (-0.92;3.43)	1.47(-0.88;3.81)	
Heart rate (bpm)	CC	92.2 ±10.2	91.2 ±9.2	-1.02 (-3.97;1.93)	-0.51(-3.40;2.39)	-0.81 (-3.87;2.25)	0.414
	CA/AA	91.4 ±9.2	92.2 ±8.9	0.76 (-2.26;3.78)	1.05(-2.04;4.14)	1.19 (-2.14;4.52)	
RSA (msec)	CC	106.6 ±53.1	109.1 ±46.2	2.46 (-12.34;17.27)	0.67 (-14.35;15.68)	2.00 (-13.86;17.87)	0.802
	CA/AA	111.5±52.2	109.0 ±53.3	-2.50 (-18.96;13.97)	-1.08 (-17.94;15.79)	0.35 (-17.85;18.55)	
PEP (msec)	CC	72.3 ±11.0	71.4 ±12.4	-0.84 (-3.98;2.30)	-1.81 (-4.95;1.33)	-1.34(-4.67;1.98)	0.431
	CA/AA	72.8 ±11.6	73.6 ±11.1	0.74 (-2.90;4.38)	0.14 (-3.54;3.83)	0.26 (-3.71;4.22)	
	**Oxytocin Polymor phism**	Maternal verbally aggressive behavior in infancy		Crude model	Model 1	Model 2	Inter-action<br><br>p-

	rs 4813627	No<br>Mean (SD)	Yes<br>Mean (SD)	Difference (95%CI)	Adjusted difference (95%CI)	Adjusted difference (95%CI)	value*
Systolic BP (mmHg)	GG	97.1 ± 8.3	97.9 ± 10.6	0.84 (-2.44;4.12)	0.14 (-3.06;3.34)	0.28 (-3.03;3.59)	0.193
	GA/AA	95.7 ± 8.0	98.8 ± 9.0	3.05 (1.07;5.03)**	2.91 (0.94;4.88)**	2.82 (0.69;4.94)*	
Diastolic BP (mmHg)	GG	57.2 ± 7.6	56.8 ± 6.7	-0.37 (-3.23;2.50)	-0.55 (-3.42;2.32)	-0.07 (-3.02;2.89)	0.554
	GA/AA	56.5 ± 7.0	57.2 ± 7.1	0.73 (-0.93;2.38)	0.64(-1.05;2.33)	0.37 (-1.46;2.20)	
Heart rate (bpm)	GG	91.9 ± 10.5	92.9 ± 10.2	0.98 (-3.38;5.35)	1.42 (-2.93;5.78)	1.48 (-3.06;6.02)	0.580
	GA/AA	91.8 ± 9.3	91.2 ± 8.6	-0.64 (-3.02;1.74)	-0.25 (-2.63;2.13)	-0.11 (-2.67;2.45)	
RSA (msec)	GG	108.2 ±55.7	100.8 ±42.0	-7.34 (-28.43;13.75)	-6.85 (-28.01;14.31)	-6.91 (-28.88;15.05)	0.530
	GA/AA	109.5±51.4	112.5 ±51.8	3.02 (-9.86;15.90)	2.20 (-10.98;15.39)	3.74 (-10.49;17.96)	
PEP (msec)	GG	71.8 ± 10.7	73.7 ± 13.6	1.96 (-2.33;6.25)	1.40 (-2.84;5.64)	1.08 (-3.35;5.51)	0.192
	GA/AA	72.8 ± 11.5	71.8 ± 11.1	-1.08 (-3.94;1.78)	-2.01 (-4.90;0.87)	-1.63 (-4.74;1.48)	

Model 1: adjusted for sex, height and age of the child; Model 2: Model 1, additionally adjusted for maternal depressive symptoms, pleasure in infant care, maternal physical aggression in infancy, authoritarian parenting style, maternal depressive symptoms and parenting stress (NOSI-K) at the child's age of 5-6.

*Interaction between maternal verbal aggressive behavior in infancy and oxytocin polymorphism *<0.05; **<0.01; ***<0.001
